# The Medical Society of the County of Erie

**Published:** 1891-08

**Authors:** B. G. Long

**Affiliations:** Secretary pro tem.


### THE MEDICAL SOCIETY OF THE COUNTY OF ERIE.

Reported by Dr. B. G. LONG, Secretary *pro tem.*

Special meeting of the Erie County Medical Society, to take action upon the death of Dr. F. H. Potter, in Association Hall, July 17, 1891.

The President, Dr. E. C. W. O'Brien, in the chair.

Dr. B. G. Long was appointed Secretary *pro tempore.*

The President, in stating the object of the meeting, paid tribute to the worth of the deceased, as follows:

*Gentlemen*—It is a sad event that brings us together tonight, meeting, as we do, to take suitable action in regard to the death of our greatly esteemed fellow-member, Dr. Frank H. Potter. Death has entered our association with grievous frequency during the past twelve months, and I am sure we feel that, in the present instance, the grim destroyer has taken one of our most highly respected and valuable members. Our deceased brother, as we well know, had accomplished a great deal that was highly creditable during his medical career. Of unusual natural abilities, which were well trained and carefully directed by his experienced and skilful father, he had won distinction to a gratifying degree in the important specialty to which he devoted his earnest attention for some years past. We knew him as a constant student, and as an untiring worker in his professional field. The well-marked recognition of his unusual talents by the local leaders of our profession must have been a source of warrantable pride and pleasure to his many friends, a fact which will, in a measure, afford consolation to those who will grieve that his earnest and useful life, that seemed so full of bright promise, has ended. His relations with his professional brethren were of the pleasantest nature, and the character of his private life was a good example for all men.

Upon Dr. Vandenbergh's motion, a committee of three upon memorial was appointed by the chair, as follows: Dr. F. W. Bartlett, Dr. F. W. Abbott, and Dr. DeLancey Rochester.

A telegram of sympathy from Dr. F. W. Hinkel was read by the Secretary.

Dr. J. Parmenter: It was my very great privilege to have known Dr. Potter intimately for some years. There is no need for me to say anything of his professional attainments; they are known to us all. But it is of his great moral worth that I like most to think and speak of him. No man of my acquaintance ever showed more loyalty to his friends, more gratitude for any little attention, more tolerance for the opinions of others, no matter how radically they differed from his own, than Dr. Potter. He took a deep, earnest view of life, was untiring in his efforts to promote good citizenship (as evidenced by his work as a civil service reformer), and assisted, as far as lay in his power, every worthy philanthropic and educational measure that was brought to his notice. He ever urged for higher education, not only in his chosen profession, but in every department of learning. He was a teacher of more than ordinary ability, as he amply demonstrated during the years he taught in the Niagara University. It was a great pleasure to me when he was elected to the chair of laryngology in the University of Buffalo, for we all felt that a distinct addition had been made to the teaching force of the college. His death is a loss that only his more intimate friends can justly estimate, for he never paraded his virtues or sang his own praise. His work did all this for him. Many of us have lost not only a colleague in whom we took pride for his energy and achievements, but a dear, loyal friend, whose life and example stood for all that is ennobling and good.

Dr. Edward Clark: I feel that I would be derelict in my duty if I should let this opportunity pass without paying my tribute of respect to the memory of my friend, Dr. Frank H. Potter. During the past ten years, my relations with Dr. Potter were so intimate, that I had ample opportunity to learn to know him well. When he first entered upon the practice of his profession here in Buffalo, we became associated together as assistant surgeons at the Buffalo Hospital of the Sisters of Charity. At that time a friendship sprang up between us, which continued unbroken to the time of his death. For the past three years we have lived side by side as neighbors, and my daily association with him afforded me unusual facilities for knowing and appreciating his sterling worth. He was modest and unassuming to a great degree, so far as his professional attainments were concerned. I regarded him as a good student and a careful observer. He was rapidly becoming an authority and an expert in his special field, and his careful and conscientious efforts in this line gave great promise of a brilliant future. To my mind the saying that "Death loves a shining mark," was never more forcibly illustrated than in the untimely death of Dr. Potter. He was a dutiful son, a fond husband and father, and while our profession will feel keenly his loss, his death falls as a crushing bereavement on those who were bound to him by nearer and dearer ties.

Dr. J.W. PUTNAM: Dr. Potter and myself were classmates at the medical college, and have been intimate friends ever since. In the course of a friendship of twelve years I have always found him a friend on whom one could lean for advice. He was a man who always gave his friends the benefit of his best judgment, never jumping at conclusions. Fair in his way of looking at questions, and fair in his dealings with men, he demanded equal fairness toward himself.

Dr. Potter was fearless in his denunciation of what was wrong, and was firm in maintaining his opinions, no matter who differed from him. He was an earnest seeker after the truth, not only of medical, but of abstract questions. His habits were those of a methodical, painstaking student. He profited by his experience, for his case records were carefully kept and studied. He had the confidence of his friends, both lay and professional, and he never abused it. He had already attained prominence in his specialty, and had he been spared he would, without doubt, have become eminent.

But Doctor Potter was not satisfied with simply doing his medical duty. He felt that the city where he lived had claims upon him, and he devoted himself to the work of Civil Service Reform. Of delicate physique, he never shrank from his work, and often after a tiresome professional day he would attend to the duty of examining candidates for the civil service appointments. Handicapped as he was by a weak physical nature, he, nevertheless, accomplished more than men who were far more favored by nature.

Personally, as a friend and companion, I have many fond recollections of him as the appreciative listener, the ready conversationalist, always willing to do a good service if it lay in his way.

Only those who knew him intimately can appreciate how much the profession has lost by losing Frank Potter.

Dr. H. D. Ingraham : Mr. President, I did not intend to say anything tonight, as I have already expressed what I had to say in regard to our late friend upon a similar occasion to this. Yet a remark made at another meeting, by a former student of Niagara University, led me to believe that Dr. Potter has left a deeper and more lasting impression upon many of the younger members of the profession than ever I supposed. This young man said that he had "listened to Dr. Potter's lectures for three years, first upon materia medica, and later upon his special branch ; that he always presented his subject clearly and concisely, often criticising methods, but never criticising individuals or speaking lightly of other members of the profession. Although he was highly respected as a teacher, it was his conduct as a gentleman that made the strongest impression upon the student." Mr. President, I believe this is true, and although Dr. Potter was a young man, he had all the finer and nobler qualities of the physician of the older school. It is a sad fact that there are too few gentlemen in the medical profession at the present time. If there were more physicians like Dr. Potter there would be less rushing into print to air supposed grievances. It must be a great satisfaction to his friends to know that although he was highly esteemed as a physician of ability, yet his strongest characteristics were his noble, gentlemanly qualities; and although his life was short, yet his students will look back upon his career as an example worthy of their best efforts to emulate.

Dr. Lucien Howe : I would add just one word to this tribute of respect to the memory of Dr. Potter. It is not because I knew him long, or well, or, indeed, always pleasantly, but it is rather because on one or two occasions his interests and mine seemed to differ in a small way, and because I think that it is possible to gain a better view of a man's character when standing in front of him, perhaps opposed to him, than that which others have who have always stood beside him. On an occasion like this it would be ungenerous in the extreme not to join my feeble voice in the universal expression of regret at his loss, for I, too, can bear witness, as perhaps every one can, to his sturdy qualities as a manly man,—generous, large-hearted, strong, and positive. Honest in his convictions, and loyal to those who were his friends, he won the admiration and esteem even of those whose opinions and interests differed at times from his own.

Dr. Potter's death creates still another vacancy in that class of bright and active younger men who began from the very first to make for themselves a place in the community and in the profession. The amount that he has accomplished by earnest work, and the example that he set to other physicians as the type of a good citizen, is a record of which many a man, much older in years, might well be proud. His early loss well illustrates the fact that

We live in deeds, not years; in thoughts, not breaths;
In feelings, not in figures on a dial.
We should count time by heart-throbs: he most lives
Who thinks the most, feels the noblest, acts the best;
And he whose heart beats the quickest lives the longest—
Lives in one hour, more than in years do some
Whose fat blood sleeps, as it slips along their veins.

Dr. HUBBELL: Ever since the organization of the Medical Department of Niagara University, in 1883, Dr. Potter has been connected with that institution, in some capacity, as teacher. At first he was assistant to the chair of surgery. Afterwards he was made Assistant in Materia Medica, and lectured for three years in that department in the regular courses. In 1886, he decided to relinquish general practice and limit his work to diseases of the nose, throat and respiratory organs. This being the case, I recommended that he be appointed to lecture on laryngology in the school. This was done, and he served in that capacity till a few weeks ago, when, in furtherance of his own interests, as he (Dr. Potter) believed, he severed his connection with Niagara, much to the regret of the faculty, to take a position in the Buffalo Medical College. In leaving the school of his first adoption, he carried with him the friendship and good wishes of every member of its faculty. As a speaker and teacher, few excelled him in systematic and clear presentation of thought. He was honorable in every respect, and he was a perfect gentleman socially. As a companion he was most genial and entertaining; as an associate he was considerate and helpful; and as a practitioner he was ambitious and progressive. He was greatly respected as a teacher. He was tenacious of his convictions, yet he made few enemies. With a high ambition to excel, with a clear and discriminating mind to guide him, and a facile pen to aid him, future years, had his life been spared, would, without doubt, have placed him among the foremost in this country in his chosen field of labor. I held the deceased in high esteem, and greatly deplored the premature cutting off of such a life. I would be glad to say more, but this is sufficient to indicate my estimate of Dr. Potter and my feelings toward him.

Dr. JOHN H. PRYOR: I have known Dr. Frank H. Potter from the bright beginning to the lamented ending of his short medical career. Our companionship was sufficiently intimate to enable me to form an opinion of his ability and rare traits of character, and I learned to admire him as a physician and prize him as a friend. He possessed natural talents of an unusual order, and a determination to excel in a profession which he honored and reverenced. Always progressive, patient, and studious, he belonged to a small band of scientific workers who appreciated his worth and knew that modest merit would place him in the front ranks in his specialty. He was a careful observer, cautious in forming an opinion, and displayed exceptionally good judgment. As a writer he was particularly successful, and his articles commanded attention by their clinical value and literary excellence. The fact that some of his contributions received flattering notice far from home, was a great source of satisfaction to him and his friends. He toiled because he loved work and had learned the secret of methodical investigation. A constitution inclined to be delicate limited the scope of his activity, but could not subdue his will, and the struggle, at times, against the harassing, fettering influence of ill-health only revealed latent qualities of resistance to trying obstacles. Although ambitious, he was true to his ideals, and the aim was so high that only slow years of conscientious effort could lead to a consummation. Of late, the way to his aspiration seemed most encouraging, and the prospect was brightening with new and cheering expectations. In the fair retrospect of his life, lofty elements of character shine in radiant prominence. Frank Potter was more than a physician ; he was a man. The beauty and purity of his moral nature was exhibited in the duties and trials of his vocation, and he represented the most elevated ideas and noblest impulses which give lustre and high meaning to a calling. Any hope for appreciation and reputation rested upon modest and unassuming merits, and confidence in the approbation gained by results. No man knew better the unwritten laws of good taste and delicate consideration. Natural elegance of manner, winning frankness, and warm sympathies, made him a most attractive and charming companion, and distinguished him as a refined gentleman in every circle. Affections were very strong with him, and tests of sincerity proved unswerving loyalty. To know him socially was a joyous experience. The product of his mind was pure and lofty, and often brilliantly enriched by literary gems from old quaint regions which he had found time to visit. He could be stern and tender. His opinions were strong, and if necessary he was fearless in advancing them. Intensely sympathetic, unselfish, finely made and keenly sensitive, he was quickly responsive in the mysterious language of feelings, and his friendship was marked by those lingering overtures of sentiments which the daily conflicts never stifled. In him were combined the essential qualities which would have more respect and commendation under any circumstances. By his death the profession suffers a double loss ; all that he had accomplished seemed but preparatory. He was regarded as a man with a future, and his early removal leaves the promises unfulfilled, The memory of Frank Potter will stir sad recollections, but they can only be expressed in words of heartfelt praise. To his friends he left all that he could — the example of a useful and beautiful life.

The committee on memorial here reported as follows :

WHEREAS, Death has removed from our ranks our esteemed fellow-member, Dr. Frank Hamilton Potter, in the morning of a professional career that gave promise of a brilliant noon :

*Resolved,* That in his death the Erie County Medical Society has lost one of its most studious, industrious and progressive members, one who in his chosen specialty had already attained an enviable position from his useful contributions to medical literature and science, and which had won for him distinguished recognition from the scientific bodies with which he had been connected.

*Resolved,* That we highly appreciate his efforts in behalf of elevating the standard of medical education.

*Resolved,* That as citizens we pay especial tribute to his valuable services in improving the municipal civil service.

*Resolved,* That in the purity, modesty, and uprightness of his character he was an example worthy of emulation.

*Resolved,*, That we tender our deepest sympathy to his bereaved family and relatives ; and that we attend his funeral in a body.

Dr. DeLancey Rochester: Mr. President, I move the adoption of the resolutions as read, and at the same time I desire to present a slight personal tribute to the worth of Dr. Potter. It has been my good fortune to have known Dr. Potter long and intimately. Our friendship began when we were but fifteen years old, and has lasted, unbroken, since. I have seen him in many trying positions and never have known him to do the wrong thing. His character was well rounded ; there was nothing small or narrow about him. He never jumped at a conclusion, but always considered every question that came before him from every side ; but having found an opinion, he had the courage of his convictions and combatted for them, through thick and thin, until he won.

In his professional work, he made many original observations, added to our knowledge of diseased conditions, and invented several instruments of great value in his special field of work. He has devoted more time than any other medical man in the city to the improvement of the municipal civil service, and to his untiring efforts in this direction is due, in large part, the successful application of the civil service reform ideas to the city government. But, among his friends and in his family, his virtues shone out preëminently, and made us love him for the purity, modesty, and unselfishness of his life. Truly, his was a beautiful character.

Dr. F. W. Abbott : Mr. President, I wish to say a very few words in memory of my friend Dr. Potter. My acquaintance with him began about ten years ago, when he was resident physician at the Rochester City Hospital. I had occasion to visit that institution, and easily recall the figure of the grave, courteous, dignified young gentleman who rose from his book to greet me as I entered. When he came to Buffalo we were not thrown together much, and I did not get well acquainted with him until he was appointed Surgeon to the Erie County Eye, Ear and

Throat Infirmary a few months ago. There I met him frequently, and learned to esteem him highly for his personal character and professional ability. At the meetings of the staff, while courteous and deferential to his seniors, he was ready with well-considered plans and ideas of his own for the management of the institution. I looked forward with pleasure to years of intimate association in this work with so pleasant and accomplished a gentleman, but he has been taken from us, and we deeply mourn his loss.

Drs. Irving M. Snow and John Cronyn also spoke very feelingly to the memory of Dr. Potter.

A motion to adopt and transmit copies to the papers and family by Dr. Rochester, was seconded by Dr. Putnam. Carried.

---

### ACTION OF THE FACULTY OF NIAGARA UNIVERSITY.

Special meeting of the faculty of the Medical Department of Niagara University, held in the faculty rooms, July 17, 1891.

The President, Dr. Cronyn, stated the object of the meeting to be to take action on the death of our late associate, Dr. Frank Hamilton Potter. In fitting terms the noble qualities of Dr. Potter were enumerated—his many virtues, universal friendship and gentlemanly bearing made him honored and beloved by all.

Dr. Lothrop paid a high tribute to his memory, and moved that a committee be appointed to draw up a fitting memorial.

Drs. Lothrop, Hubbell and Krauss were appointed by the President to serve on the committee.

In the meantime the following members of the faculty availed themselves of the opportunity to speak personally of their acquaintance with Dr. Potter, as follows:

Dr. H. D. Ingraham.—*Mr. President:* You have just remarked that while the committee are out it is proper for those who remain to say anything that we wish in regard to our late friend, which you think will be in his praise, as you believe nothing but good can be said of him. I fully agree with you, Mr. President. I cannot say anything here or elsewhere of Dr. Frank H. Potter except what is good. I know nothing about him but what is good. I have been quite well acquainted with him for the past eight years. I have met him in our deliberations as a faculty, as a teacher, at the hospital, and in various other professional and social relations, and have always found him an able physician and an honest, honorable man. Since he went into special practice he achieved an enviable position in a very short time, one that many older men might be proud of. As an honest, honorable physician and a man he was certainly the equal of any and the superior of most of the profession, always treating his professional brother as he wished to be treated.

Dr. J. A. Miller.—*Mr. President:* Words are meaningless in giving expression to my feelings on this occasion. Ever since my return to Buffalo Dr. Potter has been one of my warmest friends, and I can say that I found him a *true friend*, indeed. He was a man whose purity of soul, honesty and sincerity of purpose, nobility and manliness of character, endeared him to all who knew him.

Dr. Persons: I heartily concur in all that has been said concerning the private and professional life of our late friend and colleague, Dr. Potter. My acquaintance with him began soon after I came to Buffalo, eight years ago, and my admiration for his many noble qualities and excellent attainments increased each year as we became more intimate. The whole expression of his life was one of purity and of noble purposes; the mainspring of his ambition centered in a desire to benefit and elevate his fellow-man.

In the many confidential talks we had regarding our college work, his greatest aspirations seemed to be in the interests of a higher medical education, and the hope that the medical profession would soon appreciate the efforts of his colleagues in its behalf. His loyalty to his friends, and to the institution with which he had been so long connected, cannot be questioned, and his most recent utterances served the more strongly to assure us of his good wishes.

I can only deplore the fate that has taken from our midst a man whom we all loved and honored, whose natural gifts and training would have eventually placed him in the front rank of his chosen profession. His estimate of character was generally very near the truth, and his charity for others' weakness afforded an example which all could emulate with profit.

In the untimely death of Frank Hamilton Potter the profession has lost one of its best exponents of "the gentleman in medicine."

Dr. Hubbell: Dr. Potter, having been connected with this school from the early part of its organization till, much to our regret, he tendered his resignation a few weeks ago, it is most fitting that we should, as a body, pay our respects to his memory. The presence, today, of nearly every member of our faculty who is not absent from the city, attests, if in no other manner, the esteem and regard in which he was held by us.

The memorial which the committee, through its chairman, Dr. Lothrop, has presented, truthfully, and I believe unanimously, voices the sentiments of this faculty. What I would desire to say would largely be but a repetition of them. I wish to add, however, my personal tribute to his character and memory. The social, and especially the college, relations between Dr. Potter and myself have been more or less intimate for several years. When I learned that he intended to relinquish general practice, in 1886, and pursue special work in diseases of the nose and throat, I invited him, upon the sanction of the faculty, to teach the senior students in this department, which the trustees of the university had assigned to me. He had already proved himself an apt and clear teacher, and in this new and preferred field he still further distinguished himself in the estimation of those who were the recipients of his instruction. Another proof of his success as a teacher was the uniformly high percentage which students, each year, acquired in his examinations.

In his relations with this faculty Dr. Potter was always upright and courteous. His knowledge of men, his clear insight, his acute sense of discrimination, coupled with honor, culture, and excellent professional attainments, made his presence desirable and his opinions of weight in the deliberations of this faculty. He possessed the qualities of character and loftiness of purpose which, I believe, would have made for him a wide reputation in his specialty, could life and health have been spared. Dr. Potter's death is a serious loss to the community, to the profession, to a doting household, and a personal loss to me.

Dr. Geo. Fell: I have been acquainted with Dr. Potter for some years previous to his association with the faculty of this college. My relations with him have been such that I had the fullest respect for his manly, upright character. There was something inexpressibly sad in the blotting out of a life just beginning to bud freely into the successes and joys which follow the trials always experienced, particularly in the medical profession, before honest success is attained. Professionally successful, the future offered hopes most encouraging. A young, happy family growing up. What more could be asked? And yet this life is striken down just when it appeared to be most needed to fill out the meed of hopeful, expectant satisfaction to a large circle of relatives and friends. The problems surrounding our lives are, indeed, from a rationalistic standpoint, unexplainable. I respected Dr. Potter, and I sympathize with those who mourn his loss, and came here to express my feelings.

Dr. H. C. Buswell: I was privileged to listen to Dr. Potter's lectures for three years, and can, therefore, speak intelligently of him as a lecturer. He never addressed a class without preparation, and, being thoroughly conversant with the subject, spoke readily, and presented the subject-matter concisely and logically, making it easy for the student to comprehend and remember. As a lecturer and teacher he had few superiors in the college.

But that for which I honored him most was the fact that he was an educated, professional *gentleman*. Deplorable as it may be, there undoubtedly exists a class of medical men, who, under the false color of progression, are nothing more nor less than medical hucksters, whose self-laudation and solicitation for trade are only limited by their vocal capacity and the versatility of their bosom friends, the reporters of the penny papers

In direct contrast to these methods we have seen our lamented friend, in a legitimate and honorable manner, acquire substantial practice and high reputation. His private and professional conduct were above reproach. Had we more like him, codes of ethics and votes of censure would be less needful.

I hold sacred the memory of Frank Hamilton Potter because of his ability, integrity, and honor.

Dr. William C. Krauss.—I do not wish to let this occasion pass without paying my tribute to the memory of our late colleague and associate, Dr. Frank Potter. It is now about two years since I first met him, and since then we have been thrown together socially and professionally, so that I had learned to esteem and honor him highly as a friend and a gentleman. It was a pleasure to be in his company; and in the many faculty, journal, and society meetings which we have attended together, I have more and more been impressed with his qualities as a just and conscientious leader. Among the younger men of our profession he was sought for his opinions, and we always found him squarely on one side, and that side proved to be the right side.

I regretted very much when I heard of his intent to leave us, because men of his intelligence and judgment go far towards making a thorough and illustrious faculty. His name and fame had extended beyond the city limits, and I doubt not but that in a few years he would have been regarded as a high authority in his chosen specialty. His death we all mourn, more so, perhaps, because it came without warning; his memory will ever be dear to us.

Dr. S. A. DUNHAM: I believe it the sentiment of every student of this university that Dr. Frank Hamilton Potter was highly esteemed and respected as a teacher, and his personal friendship was inestimable. Socially and professionally his acquaintance was very gratifying and helpful to me. His early success and popularity was certainly due to his integrity of character and earnestness of purpose.

The committee on memorial reported as follows:

The Faculty in Medicine of Niagara University, assembled, at the call of its President, to give expression to its sentiments on the death of our late associate, Frank Hamilton Potter, M. D., late Lecturer on Laryngology in this College, desire to record their sorrow at the Divine dispensation which takes from earth a public-spirited citizen, from the medical profession an accomplished member, and from his family circle a devoted son and brother, and a loving husband and father.

We record with pride his success and influence as a teacher in this School. The uniform testimony of the senior students, to whom he lectured, is expressed by his popularity among them, and in the thorough comprehension of the subject he taught among his auditors.

In the specialty to which he consecrated his energy and skill, the success of his labors is attested by the concurrent testimony of the profession and his patients. The assiduous study and work, and eminent ability which he brought to his profession, were soon to be recognized and rewarded by an honor, which he cherished and merited, in the election to membership in the National Association of Laryngologists.

Beyond his profession, he had already laid up a fund of intellectual knowledge which gave him recognition in the social and learned circles of this city.

In his daily walk and conversation he was singularly pure, and honorable and, upright, respected by all, and devotedly loved by his intimate friends.

Such a life, suddenly brought to a close at an early age, when he was soon to enter upon a career of professional success for which he had long and diligently struggled, is another example where death selects a shining mark, interrupting the execution of matured plans and blighting honorable aspirations, which would have led to a distinguished position in his chosen specialty.

This Faculty beg to convey to the afflicted family assurances of our deepest sympathy in their bereavement.

It is also directed that the members of this Faculty attend the funeral services of our late associate in a body.

It is further directed that this memorial be engrossed, and the Secretary transmit the same to the family of the deceased.

Respectfully submitted,

THOS. LOTHROP,
A. A. HUBBELL,
WM. C. KRAUSS,
*Committee.*

MEDICAL DEPARTMENT, NIAGARA UNIVERSITY, July 17, 1891.

---

### ACTION OF THE FACULTY OF BUFFALO UNIVERSITY.

Reported by DR. IRVING M. SNOW, Secretary.

A SPECIAL meeting of the faculty of the University of Buffalo, Medical Department, was held Friday evening, July 17th, Dr. LUCIEN HOWE presiding, and Dr. IRVING M. SNOW acting as Secretary.

Dr. HOWE announced the death of Dr. Frank H. Potter, Clinical Professor of Laryngology, and said that it was fitting that the faculty take some action upon the loss of so talented a colleague.

A committee of three was appointed to draft resolutions, consisting of Drs. Putnam, Parmenter and Rochester.

Dr. BARRETT eulogized Dr. Potter's abilities, and deplored his premature and untimely death.

Dr. A. L. BENEDICT spoke of the fatality which had followed the house surgeons of the Rochester City Hospital, four of whom had died of the same disease from which Dr. Potter died.

### REPORT OF COMMITTEE.

WHEREAS, The University of Buffalo has sustained a serious loss in the untimely death of Prof. Frank H. Potter ;

*Resolved*, That we put [illegible] record our appreciation of his high professional attainments, of his [illegible] as a teacher, of his devotion to the cause of higher medical education, [illegible] f his high ideal of the duties of citizenship, and of the purity of his c[illegible]ter.

J. W. PUTNAM,
JOHN PARMENTER,
DELANCEY ROCHESTER,
*Committee.*

The report of the committee was adopted.

Dr. PARMENTER thought Dr. Potter's pure and energetic character would have a lasting influence upon his friends.

Dr. PUTNAM spoke of his frail physique, and of the great amount of work which he accomplished, both professionally and for the cause of civil service reform.

Dr. HOWE alluded to Dr. Potter's strong character, and considered his premature death a serious loss to the younger element in the college. He spoke especially of the sympathy we all felt for his father, Dr. W. W. Potter.

Dr. ROCHESTER moved that the chair appoint three members to represent the faculty at the funeral.

Adjourned.

---

### MEMORIAL OF THE THURSDAY CLUB.

The Thursday Club, of Buffalo, at a special meeting held at the Buffalo Club, July 23, 1891, Dr. James W. Putnam being in the chair, and Dr. John Parmenter acting as Secretary, adopted the following memorial in affectionate remembrance of their lamented colleague, Frank Hamilton Potter, M. D.:

By the death of Dr. Potter the Thursday Club loses one of its founders, who, from the day of its organization to its recent adjournment for the summer vacation, was one of its most loyal and devoted members.

A man of serious purpose and strong convictions, his modest bearing, courtesy to antagonists in the heat of debate, and generous respect for the opinions of others, endeared him to his colleagues, and will preserve his name and example among the most honorable traditions of the club.

Dr. Potter was not only a charter member, but a model member, of the Thursday Club. He was a manysided man. The windows of his mind and heart were open all around, and light from every quarter was welcome. He sought the truth, and was not afraid in following it to depart from beaten paths and to stand up and be counted with the temporarily unpopular.

As a simple testimonial of their love and esteem for their late comrade, the members of the Thursday Club direct this expression of their sentiments to be sent to Dr. Potter's family, and to be made a part of the club's permanent records.

